# Sex-specific responses to mineralocorticoid receptor antagonism in hypertensive African American males and females

**DOI:** 10.1186/s13293-019-0238-6

**Published:** 2019-05-09

**Authors:** John S. Clemmer, Jessica L. Faulkner, Alex J. Mullen, Kenneth R. Butler, Robert L. Hester

**Affiliations:** 1 Department of Physiology and Biophysics, Center for Computational Medicine, University of Mississippi Medical Center, 2500 North State Street, Jackson, MS 39216-4505 USA; 2 Department of Data Sciences, John D. Bower School of Population Health, University of Mississippi Medical Center, Jackson, MS 39216-4505 USA; 3 Department of Medicine, University of Mississippi Medical Center, Jackson, MS 39216-4505 USA; 4 Vascular Biology Center, Medical College of Georgia, Augusta University, Augusta, GA 30912-5563 USA

**Keywords:** Sex-specific, Hypertension, Mineralocorticoid antagonist, African American

## Abstract

**Background:**

African Americans (AA) develop hypertension (HTN) at an earlier age, have a greater frequency and severity of HTN, and greater prevalence of uncontrolled HTN as compared to the white population. Mineralocorticoid antagonists have been shown to be very effective in treating uncontrolled HTN in both AA and white patients, but sex-specific responses are unclear.

**Methods:**

We evaluated the sex-specific impact of mineralocorticoid antagonism in an AA population. An AA cohort (*n* = 1483) from the Genetic Epidemiology Network of Arteriopathy study was stratified based on sex and whether they were taking spironolactone, a mineralocorticoid antagonist, in their antihypertensive regimen.

**Results:**

As compared to AA women not prescribed a mineralocorticoid antagonist, AA women taking spironolactone (*n* = 9) had lower systolic and diastolic blood pressure despite having a similar number of antihypertensive medications. The proportion of AA women with uncontrolled HTN was significantly less for patients taking spironolactone than for patients not prescribed spironolactone. Interestingly, none of these associations were found in the AA males or in white females.

**Conclusions:**

Our data suggests that spironolactone is particularly effective in reducing blood pressure and controlling HTN in AA women. Further research into the impact of this therapy in this underserved and understudied minority is warranted.

## Introduction

Roughly one in three American adults is hypertensive [1]. Hypertension (HTN) is the most significant predictor of coronary events, stroke, and cardiovascular-related mortality [2, 3]. General fist-line treatment therapies for HTN include angiotensin II type I receptor blockers (ARBs), angiotensin-converting enzyme (ACE) inhibitors, calcium channel blockers, adrenergic blockers, and thiazide diuretics. Despite the noted efficacy of these treatments [4–6], uncontrolled HTN remains prevalent, affecting ~ 30% of hypertensive adults [7].

African Americans (AA) develop HTN earlier and have a lower rate of controlled HTN than other ethnicities [1, 8–12]. AA characteristically develop HTN without an increase in renin-angiotensin system activity, which corresponds to a decreased efficacy of ARBs and ACE inhibitors in this ethnic group as compared to Caucasians [13–16]. Although AA typically have low-renin HTN, the ratio of circulating aldosterone:renin is elevated during HTN [17, 18]. Importantly, an elevated aldosterone:renin ratio is emerging as a prognostic tool for clinical measurement of salt-sensitive HTN, a phenotype that is particularly common in AA [19–21]. Furthermore, aldosterone levels correlate with blood pressure (BP) and vascular function in AA patients [22]. Much of the work on blocking aldosterone’s effects through mineralocorticoid receptor antagonism to treat HTN has been done in populations that were predominantly white males [23, 24].

AA women are more likely to develop salt-sensitive HTN as compared to both AA men and white women, a manifestation characterized by an elevated aldosterone:renin ratio [19–21, 25]. In addition, in a predominantly AA population, obese women have a greater BP lowering response to mineralocorticoid receptor antagonists [26]. Experimental data, from obese mouse models, suggests that mineralocorticoid receptor antagonists ameliorate obesity-associated vascular dysfunction, diastolic dysfunction, and HTN in female, but not male mice [27–29]. Therefore, in the current study, we hypothesized that spironolactone, a mineralocorticoid receptor antagonist, has sex-specific efficacy in a cohort of AA patients. An additional analysis was done on white females to assess race specificity.

## Methods

### Study population

The Genetic Epidemiology Network of Arteriopathy (GENOA) study is one of four networks in the Family Blood Pressure Program investigating cardiac and renal complications of HTN in whites and AA [30]. This study was designed to be a multicenter community-based study to identify genes influencing BP and the development of target organ damage due to HTN. The AA participants were recruited from Jackson in Hinds County, Mississippi, while the non-Hispanic white participants were recruited from Rochester in Olmsted County, Minnesota. From 1996 to 2000 (phase I), sibships with at least 2 individuals diagnosed with essential HTN prior to the age of 60 years were enrolled in Jackson (AA subjects, *n*  =  1854) and Rochester (white subjects, *n*  =  1583). From 2001 to 2004 (phase II), these participants returned for a second study visit and underwent a physical examination, provided blood samples and underwent characterization of subclinical markers of arteriosclerosis. All data presented in the current study are from phase II. The GENOA study was approved by the Institutional Review Board at both institutions (University of Mississippi Medical Center and Mayo Clinic), and all participants gave written informed consent.

Blood samples were collected into Vacutainers with ethylenediaminetetraacetic acid (Becton Dickinson, Franklin Lakes, NJ, USA) for determining plasma glucose and creatinine in the Mayo General Clinical Research Center Immunochemical Core Laboratory. The Mayo Clinic Quadratic equation was used to estimate glomerular filtration rate (eGFR) using plasma creatinine. Standardized echocardiography was performed by trained field-center technicians at New York Presbyterian Hospital-Weill Cornell Medical Center.

### Measurements of covariates

Demographics and medical history were collected from standard questionnaires. Height was taken from a stadiometer, and weight was measured by electronic balance to calculate body mass index (BMI). Resting systolic and diastolic blood pressure (SBP and DBP, respectively) were measured by an automated BP monitor (Dinamap, Critikon Corporation, Tampa, FL, USA). BP was averaged from three measurements that were taken from the right arm after being seated for at least 5 min.

Classification of HTN was based on a definitive clinical diagnosis by a physician based on SBP ≥ 140 or DBP ≥ 90 mmHg. The proportion of individuals with uncontrolled HTN was defined as individuals with HTN not being controlled (SBP ≥ 140 or DBP ≥ 90 mmHg). Diabetes classification was based on fasting serum glucose ≥ 126 mg/dL or existing oral hypoglycemic medication. BP medication use and previous medical history such as stroke or coronary heart disease (CHD) were obtained from questionnaires completed by the participants. CHD was defined as previous myocardial infarction (MI), coronary artery bypass surgery, or angioplasty [31]. Mineralocorticoid receptor antagonist use was indicated by patient medicine regimens that included oral prescriptions of spironolactone or eplerenone. Dosage and frequency were not reported.

### Statistical analysis

A total of 2759 participants (1518 from Jackson, MS, USA, and 1241 from Rochester, MN, USA) completed both GENOA visits. Of the 1482 AA individuals with echocardiography data, there were 1446 AA patients screened with definitive normotensive/HTN diagnoses and 1406 of these individuals had no evidence of heart failure (ejection fraction > 40). None of the white participants had echocardiography data. Because only 3 white males were taking a mineralocorticoid antagonist, this group was excluded from this study. Direct statistical comparisons between AA and white groups were not performed. There were 684 white female individuals with definitive normotensive/HTN diagnoses who were included in the analysis. Data were summarized using proportions for categorical variables and means (standard deviations) for quantitative variables. Chi-squared tests were used for comparison across categories (prevalence %). Non-dichotomous data were analyzed with ANOVA for normally distributed variables. When assumptions of normality were not justified, the data were also analyzed with the nonparametric Mann-Whitney test, as appropriate. All statistical analyses were performed using GraphPad Prism 5 (La Jolla, CA, USA). Probability was based on two-tailed tests of significance, and significance was considered *P* < 0.05.

## Results

AA patients were classified into 6 groups: male normotensive patients (AA MN, *n* = 92), male HTN patients not taking spironolactone (AA MH, *n* = 301), male HTN patients taking spironolactone (AA MH + S, *n* = 9), female normotensive patients (AA FN, *n* = 172), female HTN patients not taking spironolactone (AA FH, *n* = 823), and female HTN patients taking spironolactone (AA FH + S, *n* = 9). For additional comparisons, there were *n* = 684 white female patients; of which, *n* = 182 were normotensive (white FN), *n* = 490 with HTN (white FH), and *n* = 12 individuals with HTN who were also treated with spironolactone (white FH + S). Note that each patient’s data was collected at a single time point. No follow-up measurements were reported.

### HTN with or without spironolactone in AA patients

**Fig. 1:**
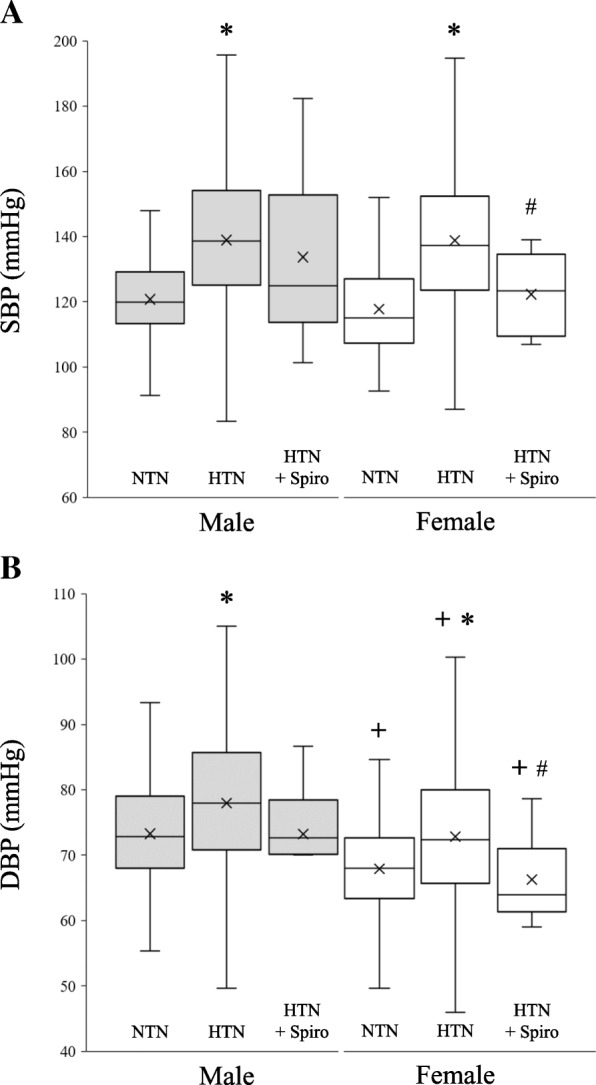
Systolic and diastolic pressures from African American males (dark) and females (light) with normal blood pressure (NTN), hypertension (HTN), or hypertension with spironolactone included in their antihypertensive regimen (HTN + Spiro) shown as box-whisker plots. *p < 0.05 vs. NTN, #*p* < 0.05 vs. HTN, +*p* < 0.05 vs. male

Patients classified as hypertensive had significantly greater BP than did all normotensive counterparts (Fig. 1, Table 2). Figure 1 demonstrates the SBP and DBP for the 6 AA groups. AA MH + S patients showed no significant SBP or DBP differences compared with AA MH patients (SBP 134 ± 26 vs. 139 ± 22 mmHg; DBP 73 ± 8 vs. 78 ± 11 mmHg). However, AA FH + S patients showed significantly lower SBP (122 ± 26 vs. 139 ± 22) and DBP (66 ± 6 vs. 73 ± 10) as compared with AA FH patients.

Baseline demographics, laboratory data, and prevalence of comorbid conditions are shown in Table 1. AA patients had a BMI of 32 ± 7 kg/m^2^ with an age of 63 ± 9 years. In AA females, compared with HTN, those taking spironolactone did not have significantly different age, BMI, number of antihypertensive medications, eGFR, or left ventricular (LV) mass. However, as compared to AA FH, FH + S patients did have significantly higher HTN duration, lower total urinary protein, and a significantly less proportion with uncontrolled HTN (Table 1). Additionally, AA FH + S had a statistically similar prevalence of CHD (11% vs. 9%) and myocardial infarction (11% vs. 4%, *p* = 0.3) as compared to AA FH. As compared to their female counterpart, AA MH patients showed significantly greater prevalence of CHD, MI, and stroke (Table 1). In AA, HTN groups also had significant increases in LV mass (Table 1). In AA FH + S, there were similar stroke volume (79 ± 14 vs. 77 ± 14, *p* = 0.7), ejection fraction (60 ± 5 vs. 62 ± 6, *p* = 0.1), and cardiac output (5237 ± 681 vs. 5195 ± 1190, *p* = 0.6) as compared to AA FH (not shown). In the entire cohort, HTN was associated with a significantly higher prevalence of CHD, MI, stroke, diabetes, increased age, greater BMI, and lower eGFR (Tables 1 and 2).

**Table 1:** Characteristics and prevalence of comorbid conditions in African American males and females

Variable	Men (*n* = 402)			Women (*n* = 1004)		
	NTN (*n* = 92)	HTN (*n* = 301)	HTN + Spiro (*n* = 9)	NTN (*n* = 172)	HTN (*n* = 823)	HTN + Spiro (*n* = 9)
Age (year)	58 ± 9	65 ± 9*	63 ± 9	56 ± 10	64 ± 9*	68 ± 9*
BMI (kg/m^2^)	28 ± 5	29 ± 5*	34 ± 6*^#^	31 ± 7^+^	33 ± 7*^+^	33 ± 4
HTN medications	0	1.7 ± 1.1*	2.7 ± 1.3*^#^	0	1.7 ± 1.1*	1.8 ± 0.8*
HTN duration (year)	0	15 ± 12*	15 ± 11*	0	17 ± 13*	29 ± 11*^#+^
eGFR (ml/min)	104 ± 17	87 ± 24*	93 ± 14*	96 ± 10^+^	87 ± 14*	79 ± 16*
LV mass (g/m^2^)	75 ± 14	90 ± 22*	90 ± 7*	68 ± 12^+^	80 ± 18*^+^	73 ± 10+
Total urinary protein (mg)	4 ± 7	16 ± 44*	11 ± 10*	4 ± 6	12 ± 34*	3 ± 3^#+^
Uncontrolled HTN (%)	0	48*	44*	0	46*	0^#+^
CHD (%)	2	17*	22*	1	9*^+^	11*
MI (%)	0	10*	22*	1	4*^+^	11*
Stroke (%)	1	10*	22*	1	5*^+^	0
Diabetes (%)	10	30*	11	8	31*	33*

*NTN* indicates normotension, *HTN* hypertension, *HTN + Spiro* hypertension with spironolactone treatment, *BMI* body mass index, *HTN* hypertension, *eGFR* estimate glomerular filtration rate, *LV* left ventricle, *CHD* coronary heart disease, *MI* myocardial infarction. *p < 0.05 vs. NTN, #*p* < 0.05 vs. HTN, +*p* < 0.05 vs. men

**Table 2:** Characteristics and prevalence of comorbid conditions in white females

Variable	NTN (*n* = 182)	HTN (*n* = 490)	HTN + Spiro (*n* = 12)
Age (year)	51 ± 9	61 ± 9*	58 ± 11*
BMI (kg/m^2^)	29 ± 7	32 ± 7*	34 ± 5*
SBP (mmHg)	120 ± 13	136 ± 20*	132 ± 24*
HTN medications	0	1.6 ± 0.9*	1.7 ± 0.8*
HTN duration (year)	0	14 ± 12*	22 ± 14*
eGFR (ml/min)	100 ± 8	90 ± 13*	90 ± 11*
Total urinary protein (mg)	2 ± 2	2 ± 4	2 ± 1
Uncontrolled HTN (%)	0	42*	25*
CHD (%)	1	11*	25*
MI (%)	0	5*	0
Stroke (%)	1	3*	8*
Diabetes (%)	3	13*	42*^#^

*NTN* normotension, *HTN* hypertension, *HTN + Spiro* hypertension with spironolactone treatment, *BMI* body mass index, *HTN* hypertension, *eGFR* estimate glomerular filtration rate, *LV* left ventricle, *CHD* coronary heart disease, *MI* myocardial infarction. *p < 0.05 vs. NTN, #*p* < 0.05 vs. HTN

### Normotensive vs. HTN and male vs. female

In both male and female AA, HTN groups had significantly increased total urinary protein as compared to normotension (Table 1); however, this difference was not significant in white females (Table 2). As compared to AA female counterparts, AA males consistently had greater LV mass (Table 1), end diastolic volume, and cardiac output (not shown). In AA HTN groups, males also consistently had a greater prevalence of CHD, MI, and stroke as compared to females (Table 1).

### HTN with or without spironolactone in white female patients

Baseline demographic and laboratory data for white females are shown in Table 2. White females did not have echocardiographic data. As compared to white FH patients, white FH + S patients did not have significantly different age, BMI, eGFR, duration of HTN, or number of HTN medications (Table 2). Unlike AA females, white FH + S patients showed no significant differences in SBP (132 ± 24 vs. 136 ± 20, *p* = 0.6), DBP (70 ± 12 vs. 73 ± 10, *p* = 0.5), or prevalence of uncontrolled HTN (25% vs. 42%, *p* = 0.2) compared with white FH patients that were not on spironolactone. FH + S patients did have a significantly higher incidence of diabetes as compared to FH individuals (Table 2).

## Discussion

The major findings of this study were that in older hypertensive patients, (1) AA women treated with spironolactone were better controlled than AA women who were on different antihypertensive regimens, and (2) while AA females received benefit from spironolactone, spironolactone treatment appeared to have no BP-lowering effect in AA men or white females.

As compared to AA MH, AA MH + S used significantly more antihypertensive medications on average but had no significant difference in SBP and had a statistically similar proportion of uncontrolled HTN (Table 1). FH + S patients were taking a similar number of medications as FH patients but still had significantly better BP control (~ 16 mmHg lower) and lower incidence of uncontrolled HTN (Fig. 1, Table 1). White females on spironolactone (white FH + S) appeared to have no benefit as compared to white FH (Table 2). This data suggests a sex-specific benefit, particularly in AA, from this antihypertensive therapy and could help further patient-specific medicine in this group of older (~ 65 years) hypertensive women.

While there were no significant BP associations in AA males or white females with spironolactone use, this may have been due to small sample sizes. Mineralocorticoid receptor antagonists have been well characterized and shown to be superior in BP control as compared to other drugs in both white and black populations [11, 32–34]. Spironolactone has greater efficacy in patients with higher sodium diets [35] or with lower renin levels [11, 36] and lowers BP in white populations, regardless of age or gender [37]. In a cohort of white patients with HTN, spironolactone lowered SBP ~ 4 mmHg more than other antihypertensive drugs [36]. Comparatively, a lower SBP (4–6 mmHg) was also found in both AA males and white females in the current study (Fig. 1, Table 2), although not reaching statistical significance. Spironolactone has also been shown to have significant BP-lowering effects in the black population [11, 33]. Recently, in diabetic African patients (mostly female) with resistant HTN and renal disease, spironolactone significantly reduced SBP (~ 30 mmHg), but sex differences were not reported [33].

Specific race and sex differences in the response to mineralocorticoid antagonists have not been well characterized. Comparing AA with whites, most clinical studies have found statistically similar reductions in BP with spironolactone [11, 34, 38]. However, in a population of mostly hypertensive AA, females tended to have a ~ 6 mmHg greater fall in SBP in response to spironolactone as compared to males, but this did not reach significance [11]. These data are similar to another study demonstrating obese females have a greater BP reduction with mineralocorticoid antagonism as compared to men, in a primarily type 2 diabetic, AA population [26]. Our data showing AA FH + S have significantly lower BP as compared to AA females on a different antihypertensive regimen (AA FH) support these findings and warrant further investigation. These data suggest that spironolactone treatment may be more efficacious in certain populations, including female AA and patients with high-sodium intake and low-renin levels.

It is important to note that the clinical population observed in the current study is a predominantly obese population. Obesity is a significant contributor to HTN and is believed to be an underlying risk factor in most hypertensive patients [39–43]. While both obese men and women are more likely to develop HTN and cardiovascular diseases, emerging experimental and clinical data suggests that the contribution of obesity to HTN is sex-specific [44]. In particular, the contribution of obesity to BP is more pronounced in women, including AA populations, as evidenced by a more closely associated BP increase per unit of BMI in women than in men [8, 45–47]. This is especially impactful in AA women who tend to have high rates of obesity [48]. Aldosterone levels are positively correlated with BMI [49, 50], a relationship that is stronger in women than in men [51]. Furthermore, experimental data indicates that obesity-associated BP increases and vascular dysfunction in female animal models is mediated by mineralocorticoid receptor activation [27–29]. Interestingly, in the current study, AA MH + S had similar BMI as compared to their female counterpart, but spironolactone treatment was not associated with a significant benefit in BP or BP control (Table 1). Additionally, white FH + S had a statistically higher prevalence of diabetes and little to no benefit in BP control as compared to white FH, as opposed to the significant BP reductions reported in AA females with type 2 diabetes [26]. These data suggest that, in females, HTN is significantly ameliorated in our predominately obese AA HTN cohort by mineralocorticoid receptor blockade. This may due to sex differences in obesity-derived BP elevation mechanisms, a deduction that warrants further study.

### Perspectives

Mineralocorticoid receptor antagonists, the primary derivatives being spironolactone and eplerenone, are used to treat hypokalemia, heart failure, acne, and polycystic ovary syndrome in addition to hypertensive indications. However, these drugs are generally reserved for patients with advanced-stage resistant HTN. These restrictions may have contributed to the small cohort size. In the current study, the average patient taking spironolactone was on ~ 2 antihypertensive medications and over the age of 60. This postmenopausal age of AA females should have deleterious effects on BP control as compared to male counterparts [52], making the current findings stronger. Nevertheless, despite little data available, our data agrees with other evidence that mineralocorticoid receptor antagonism may be particularly beneficial in older AA females with HTN [26]. Further research is needed to identify these mechanisms that play a role in this population and on the patient-specific efficacy of this drug earlier in the progression of HTN.

### Limitations

The results from the current study are exciting but are far from definitive. The need for follow-up studies and prospective clinical trials to confirm the race and sex-specific benefits of mineralocorticoid receptor antagonists is clear. One of the primary limitations of the current study is the low number of patients taking spironolactone. Future studies using a larger population, in addition to controlled clinical trials, are needed in to investigate the underlying mechanisms of mineralocorticoid receptor antagonists in AA. Another limitation of this study is that each patient’s data was collected at a single time point. Thus, the patient’s status before antihypertensive therapy is not known, and their physiological responses to spironolactone or the duration of spironolactone use is unknown. Spironolactone use during heart failure can skew the interpretation of BP, especially when ejection fraction is compromised, tending to decrease BP. AA with suspected heart failure (ejection fraction < 40) were removed from the analysis. However, white females on spironolactone had no echocardiography data to confirm the absence of heart failure as was done with the AA cohort. Even if this was a confounding factor in the white female cohort, this was not reflected in their BP or the proportion with uncontrolled HTN. Finally, direct comparisons between AA and white females were not performed due to insufficient power for comparing the spironolactone groups and due to possible confounding effects of geographic and socioeconomic factors.
